# Leukocyte Associated Immunoglobulin Like Receptor 1 Regulation and Function on Monocytes and Dendritic Cells During Inflammation

**DOI:** 10.3389/fimmu.2020.01793

**Published:** 2020-08-19

**Authors:** Tiago Carvalheiro, Samuel Garcia, M. Inês Pascoal Ramos, Barbara Giovannone, Timothy R. D. J. Radstake, Wioleta Marut, Linde Meyaard

**Affiliations:** ^1^Center for Translational Immunology, University Medical Center Utrecht, University of Utrecht, Utrecht, Netherlands; ^2^Department of Rheumatology & Clinical Immunology, University Medical Center Utrecht, University of Utrecht, Utrecht, Netherlands; ^3^Rheumatology & Immuno-Mediated Diseases Research Group (IRIDIS), Galicia Sur Health Research Institute (IIS Galicia Sur), SERGAS-UVIGO, Vigo, Spain; ^4^Rheumatology Department, University Hospital Complex of Vigo, Vigo, Spain; ^5^Oncode Institute, Utrecht, Netherlands; ^6^Department of Dermatology, University Medical Center Utrecht, Utrecht University, Utrecht, Netherlands

**Keywords:** LAIR-1, CD305, monocytes, macrophages, dendritic cells, inflammation

## Abstract

Inhibitory receptors are crucial immune regulators and are essential to prevent exacerbated responses, thus contributing to immune homeostasis. Leukocyte associated immunoglobulin like receptor 1 (LAIR-1) is an immune inhibitory receptor which has collagen and collagen domain containing proteins as ligands. LAIR-1 is broadly expressed on immune cells and has a large availability of ligands in both circulation and tissues, implicating a need for tight regulation of this interaction. In the current study, we sought to examine the regulation and function of LAIR-1 on monocyte, dendritic cell (DC) and macrophage subtypes, using different *in vitro* models. We found that LAIR-1 is highly expressed on intermediate monocytes as well as on plasmacytoid DCs. LAIR-1 is also expressed on skin immune cells, mainly on tissue CD14^+^ cells, macrophages and CD1c^+^ DCs. *In vitro*, monocyte and type-2 conventional DC stimulation leads to LAIR-1 upregulation, which may reflect the importance of LAIR-1 as negative regulator under inflammatory conditions. Indeed, we demonstrate that LAIR-1 ligation on monocytes inhibits toll like receptor (TLR)4 and Interferon (IFN)-α- induced signals. Furthermore, LAIR-1 is downregulated on GM-CSF and IFN-γ monocyte-derived macrophages and monocyte-derived DCs. In addition, LAIR-1 triggering during monocyte derived-DC differentiation results in significant phenotypic changes, as well as a different response to TLR4 and IFN-α stimulation. This indicates a role for LAIR-1 in skewing DC function, which impacts the cytokine expression profile of these cells. In conclusion, we demonstrate that LAIR-1 is consistently upregulated on monocytes and DC during the inflammatory phase of the immune response and tends to restore its expression during the resolution phase. Under inflammatory conditions, LAIR-1 has an inhibitory function, pointing toward to a potential intervention opportunity targeting LAIR-1 in inflammatory conditions.

## Introduction

Inflammation is a normal physiological response of the immune system to a variety of factors, including pathogens, damaged tissue, malignant cells, and toxic compounds. Under normal circumstances, inflammation rapidly ends to prevent adverse events. However, an exacerbated inflammatory response may result in autoimmunity and unwanted collateral damage or immune pathology (1, 2). Uncontrolled inflammation is a key player in the pathogenesis of many chronic conditions and a persistent inflammatory response can lead to significant tissue and organ damage (3, 4). Inhibitory immune receptors are essential for immunological homeostasis; during health, immune responses are balanced to prevent damage to self, while being aggressive enough to eliminate pathogens and tumors (5).

Leukocyte associated immunoglobulin-like receptor-1 (LAIR-1), also known as CD305, is a transmembrane glycoprotein inhibitory receptor with a cytoplasmic tail containing two immunoreceptor tyrosine-based inhibitory motifs (ITIMs) (6, 7). LAIR-1 has previously been shown to be expressed on almost all immune cells, including NK cells, T cells, B cells and monocytes, monocyte derived dendritic cells (moDCs), eosinophils, basophils and mast cells, as well as on CD34^+^ hematopoietic progenitor cells, the majority of thymocytes, but also neutrophils upon activation (7, 8).

Collagens are functional LAIR-1 ligands and directly inhibit immune cell activation *in vitro* (9). In addition, LAIR-1 also recognizes proteins that have collagen domains, such as surfactant protein D (10) and C1q, a component of the classical complement pathway (11). Activation of LAIR-1 *in vitro* potently inhibits diverse immune functions. Crosslinking of LAIR-1 results in inhibition of T cell receptor-mediated signaling (12–14), immunoglobulin (Ig)G and IgE production by B cells (15) and lysis of target cells by NK cells (6). Moreover, LAIR-1 crosslinking and C1q stimulation suppresses interferon alpha (IFN-α) release in plasmacytoid dendritic cells (pDC) (11, 16) and toll-like receptor (TLR)9-stimulated cytokine production by monocytes (17).

Aberrant LAIR-1 expression has been associated with autoimmune diseases, leukemia and viral infections. For example, pDCs and B cells from SLE patients express lower levels of LAIR-1, resulting in increased IFN-α and antibody secretion upon stimulation (16, 18). Moreover, soluble LAIR-1, a shed form of LAIR-1, and the soluble family member LAIR-2 are increased in urine and synovial fluid of rheumatoid arthritis patients (19). Additionally, LAIR-1 is absent in high–risk B cell chronic lymphocytic leukemia cells and LAIR-1 is downregulated on NK cells isolated from patients enduring a chronic active Epstein-Barr virus infection (20, 21). More recently, it was shown that LAIR-1 is expressed on *in vivo* activated human neutrophils and that LAIR-1 suppresses neutrophil extracellular trap formation by airway-infiltrated neutrophils obtained from patients with respiratory syncytial virus (RSV) bronchiolitis (22). In mice, LAIR-1 limits neutrophilic airway inflammation (23).

LAIR-1 is a distinctive receptor in the immune inhibitory receptor family because of the broad expression pattern of both the receptor and the ligands. The regulation of LAIR-1-mediated inhibition might be dependent on different factors such as the strength of the activation signals, the levels of expression of the receptor, but also on soluble LAIR-1 and LAIR-2 molecules (7). Potentially, the interaction of LAIR-1 with collagen could play a role in controlling immune cells in various phases of the inflammatory response. To better understand the role of LAIR-1 during inflammation, we investigated the expression and function of LAIR-1 under *in vitro* inflammatory conditions on monocyte, DC and macrophage subtypes.

## Materials and Methods

### Peripheral Blood Mononuclear Cells Isolation and Monocyte Isolation

Blood from healthy controls (HC) was obtained following institutional ethical approval. Peripheral blood mononuclear cells (PBMCs) from heparinized blood were isolated by density centrifugation using Ficoll-Paque Plus (GE Healthcare). Fresh monocytes were isolated using anti-CD14 magnetic microbeads (Miltenyi Biotec) based on positive separation on auto-MACS assisted cell sorting (Miltenyi Biotec) according to the manufacturer's protocol.

### PBMC Stimulation

A total of 1 × 10^6^ isolated PBMCs were seeded in a 48 well plate (Corning Costar) in a final volume of 0.5 mL and cultured in complete medium: RPMI 1640 GlutaMAX (Life Technologies-Thermo Fisher Scientific), supplemented with 10% heat-inactivated fetal calf serum (FCS) (Biowest Riverside) and 1% penicillin/streptomycin (Thermo Fisher Scientific). Cells were left unstimulated or stimulated overnight at 37°C in a 5% CO_2_ incubator with Pam3CSK4(P3C)-TLR2/1 ligand (5 μg/mL), LPS-TLR4 ligand (100 ng/mL), R848-TLR7/8 ligand (1 μg/mL), CpG-C-TLR9 ligand (1 μM), all from Invivogen, recombinant CXCL4 (5 μg/mL; PeproTech), recombinant IFN-α2a (1,000 U/mL, Cell Sciences), recombinant TNF-α (10 ng/mL; R&D Systems), recombinant TGF-β1 (10 ng/mL; Biolegend), and recombinant TGF-β2 (10 ng/mL; R&D Systems). Cells were then harvested and LAIR-1 expression was determined using flow cytometry. As CD141^+^ cDC1 are a very rare population in circulation and no LAIR-1 expression was detected on steady-state, no further functional experiments were performed on this cell subset.

### Skin Cells Isolation

Healthy human skin samples were collected as discarded tissue after cosmetic surgery from anonymous donors who gave prior informed consent for the use of material in research. A single-cell suspension was obtained using the whole skin dissociation kit (Miltenyi Biotech), following the manufacturer's protocol. Briefly, 3 × 4 mm biopsies were digested overnight at 37°C and processed with the gentle MACS dissociator (Miltenyi Biotech) to obtain a single cell suspension. LAIR-1 expression was determined in the single cell suspension using flow cytometry.

### Monocyte Derived Macrophage and Dendritic Cell Differentiation

Purified monocytes were cultured at a density of 1 × 10^6^ cells per mL in complete medium in the presence of recombinant GM-CSF (5 ng/mL), M-CSF (25 ng/mL), IFN-γ (10 ng/mL), IL-10 (10 ng/mL), and IL-4 (800 U/mL); all from R&D Systems, to generate macrophages. For dendritic cell differentiation, monocytes were cultured in the presence of GM-CSF (800 U/mL) in combination with IL-4 (500 U/mL), or GM-CSF (800 U/mL) in combination with IFN-α2a (1,000 U/mL, Cell Sciences). Monocytes were differentiated for 7 days at 37°C in a 5% CO_2_ incubator. At day 3, medium was refreshed with the same concentration of recombinant proteins. Cells were harvested at day 7, after 5 min incubation with accutase (Sigma-Aldrich). Next, LAIR-1 expression was determined using flow cytometry, together with the expression of CD14, CD11c, CD163, CD64, CD1a, and CD80, to assess the markers for macrophage and DC differentiation (Supplementary Figure 1).

### Flow Cytometry

Cell suspensions were first incubated with a fixable viability dye (eBioscience) to allow exclusion of dead cells and blocked either with normal mouse serum (Fitzgerald) or with Fc receptor blocking reagent (Miltenyi Biotech) and then stained for 20 min at 4°C with fluorochome-conjugated monoclonal antibodies according to the panels on Supplementary Table 1. Samples were acquired on a BD LSR Fortessa (BD Biosciences), or on a BD FACSCanto (BD Biosciences) using the BD FACSDiva software (BD Biosciences). FlowJo software (Tree Star) was used for data analyses.

### Immunofluorescence

Frozen sections (6 μm) from healthy human skin samples, collected as described above, were fixed in 4% formaldehyde for 10 min at room temperature (RT). After washing step, specimens were blocked with 5% bovine serum albumin (BSA) diluted in PBS. Next, mouse anti-human LAIR-1 biotin labeled (clone NKTA255; Abcam) or mouse isotype control IgG1 biotin labeled (eBioscience) diluted in PBS + 1% BSA buffer were incubated overnight at 4°C. Samples were then washed and incubated for 45 min with streptavidin conjugate with Alexa Fluor 594 (Life Technologies- Thermo Fisher Scientific) diluted in PBS + 1% BSA buffer. Slides were finally washed and mounted with DAPI VectaShield hardset (Vector Lab) and allow to settle before image acquisition on a Zeiss fluorescence microscopy (Zeiss) using the Axiovision software (Zeiss). Images were further processed with ImageJ software.

### Analysis of LAIR-1 Function

24 well Nunc culture plates (Thermo Fisher Scientific) were coated with 10 μg/mL of anti-LAIR-1 agonist (clone Dx26) (6) or 10 μg/mL of mouse isotype control IgG1 (eBioscience-Thermo Fisher Scientific) diluted in PBS overnight at 4°C. A total of 1 × 10^6^ PBMCs or 0.5 × 10^6^ purified monocytes were seeded in the pre-coated plates with complete medium after incubation with Fc receptor blocking reagent (Miltenyi Biotech). Cells were pre-incubated for 2 h at 37°C in a 5% CO_2_ incubator and then either left unstimulated or stimulated with LPS-TLR4 ligand (100 ng/mL, Invivogen) or IFN-α2a (1,000 U/mL, Cell Sciences). PBMCs were stimulated overnight at 37°C in a 5% CO_2_ incubator and then harvested for flow cytometry staining. Monocytes were stimulated for 5 h at 37°C in a 5% CO_2_ incubator and afterwards supernatants were collected and stored at −80°C and cells were lysed with RLT buffer (Qiagen) and stored at −20°C until further analysis.

### Crosslink of LAIR-1 During Monocyte Derived Dendritic Cell Differentiation

Purified monocytes were cultured in pre-coated 24 well plates, as described above, at a density of 1 × 10^6^ cells per mL in complete medium. To generate moDCs, recombinant human IL-4 (500 U/mL) and GM-CSF (800 U/mL); both from R&D Systems were added to the medium. moDCs were differentiated for 6 days at 37°C in a 5% CO_2_ incubator and at day 3 medium was supplemented with the same concentrations of IL-4 and GM-CSF. At day 6, cells were either harvest for flow cytometry staining or 100.000 cells were re-seeded in a 48 well culture plate (Corning, Costar) and rested overnight. On the day after, cells were left unstimulated or stimulated with LPS-TLR4 ligand (100 ng/mL, Invivogen) or IFN-α2a (1,000 U/mL, Cell Sciences) for 5 h. Finally, cells were lysed with RLT buffer (Qiagen) and stored at −20°C for further analysis.

### Measurement of Cytokine Production

Cytokines in cell-free supernatant were measured using enzyme-linked immunosorbent assay (ELISA) for IL-6 (Sanquin), IL-8 (Sanquin), TNF-α (Diaclone), CXCL10 (R&D Systems), following the manufacturer's instructions.

### RNA Isolation and Quantitative PCR

Total RNA was isolated from cell lysates using the RNeasy micro kit (Qiagen) with RNase-Free DNase Set (Qiagen), followed by retrotranscription with iScript reverse transcriptase kit (Biorad), or superscript IV (Life Technologies-Thermo Fisher Scientific) according to the manufacturer's instructions. Gene expression was determined by quantitative real-time PCR (RT-qPCR) on the QuantStudio 12 k flex (Life Technologies-Thermo Fisher Scientific) using SybrSelect mastermix (Life Technologies-Thermo Fisher Scientific) with specific primer sets listed in Supplementary Table 2. Relative gene expression levels on monocytes and moDCs were normalized using the *RPL13A* and *B2M* housekeeping genes, respectively. The relative fold change (FC) of each sample was calculated in relation to the ΔCt of the unstimulated sample treated with isotype control (reference) according to the formula FC = 2^−ΔΔCt^.

### LAIR-1 Expression From Profiling Data

LAIR-1 gene expression was retrieved from array profiling data available on the Gene Expression Omnibus (GEO–NCBI) using GEO2R (NCBI).

### Statistical Analysis

Statistical analysis was performed using GraphPad Prism 8 software (GraphPad Software Inc.). Differences between experimental groups were analyzed using parametric unpaired *t*-test, paired *t*-tests, one-way ANOVA test or non-parametric, Wilcoxon's test and Friedman test, when appropriate and corrected for multiple comparison. Pearson's correlation coefficient test was applied to detect the association between different parameters. Two-sided testing was performed for all analyses. Differences were considered to be statistically significant at *p* < 0.05.

## Results

### LAIR-1 Is Differentially Expressed on Circulating Monocytes Subsets and Dendritic Cell Subpopulations and on Skin Immune Cells

**Figure 1:**
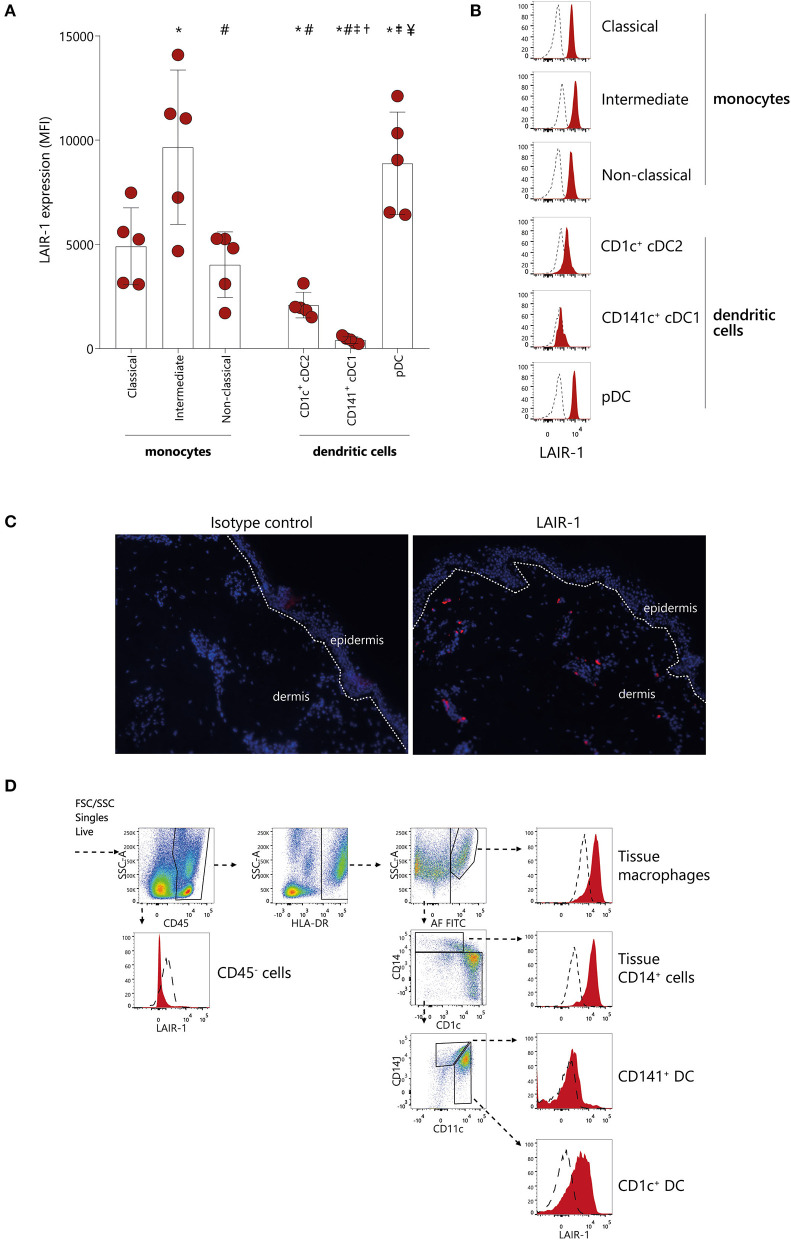
LAIR-1 is differentially expressed on circulating monocytes subsets and dendritic cells subpopulations and on skin immune cells. **(A)** Quantification and **(B)** representative histograms of LAIR-1 expression, represented as median fluorescence intensity (MFI), on classical, intermediate and non-classical monocytes as well as on CD1c^+^cDC1s, CD141^+^cDC2s, and pDCs, determined on peripheral blood mononuclear cells (PBMC) by flow cytometry. Results are represented as mean with SD. Differences were considered statistically significant when *p* < 0.05: *vs. classical monocytes, #vs. intermediate monocytes, †vs. non-classical monocytes, ^‡^vs. CD1c^+^cDC1s, ^¥^vs. CD141^+^cDC2s (one-way ANOVA test). **(C)** Immunofluorescence analysis of LAIR-1 (red staining), in normal skin section and isotype control is shown as negative control. DAPI nuclear counterstain is shown in blue. Representative images out of three independent stainings were acquired in 20 × magnification. **(D)** Flow cytometry of enzymatically digested skin. Gating strategy used to identify tissue macrophages, tissue CD14^+^ cells, CD141^+^, and CD1c^+^ DCs is shown. LAIR-1 expression (filled) on these cells is shown compared to isotype control (dashed). Representative data from three donors are shown.

LAIR-1 expression was evaluated on different monocytes subsets (classical, intermediate and non-classical) as well as on different subpopulations of classical dendritic cells (cDCs) (CD1c^+^ cDC2 and CD141^+^ cDC1) and on pDC in peripheral blood of HC (Supplementary Figure 2). LAIR-1 was expressed on all different monocyte subsets, with highest expression on intermediate monocytes, and comparable levels of expression between classical and non-classical monocytes (Figures 1A,B). Among DC subpopulations in circulation, pDC had highest levels of LAIR-1, cDC2 (CD1c^+^ DC) had intermediate levels, while cDC1 (CD141^+^ DC) did not express LAIR-1 at all (Figure 1B).

Since LAIR-1 is a collagen receptor, we next investigated whether LAIR-1 was expressed on immune cells present in collagen rich tissue. Collagen is highly present in skin, and we found that LAIR-1-expressing cells were present scattered through the dermis but not in the epidermis (Figure 1C). We next determined which cell populations expressed LAIR-1 in skin by flow cytometry, based on the populations defined by McGovern et al. (24). LAIR-1 was not expressed on non-immune cells (CD45^−^) but was highly expressed on tissue macrophages as well as on tissue CD14^+^ cells. Skin CD1c^+^ DC also expressed LAIR-1, but to lesser extent than tissue macrophages and tissue CD14^+^ cells. Similar to circulating CD141^+^ cDC1s, tissue CD141^+^ DC did not express LAIR-1 (Figure 1D). Thus, LAIR-1 is broadly expressed on blood monocytes, and it is particularly highly expressed on intermediate monocytes, and it is also expressed in skin myeloid cells.

### LAIR-1 Is Upregulated Upon Inflammatory Triggers

**Figure 2:**
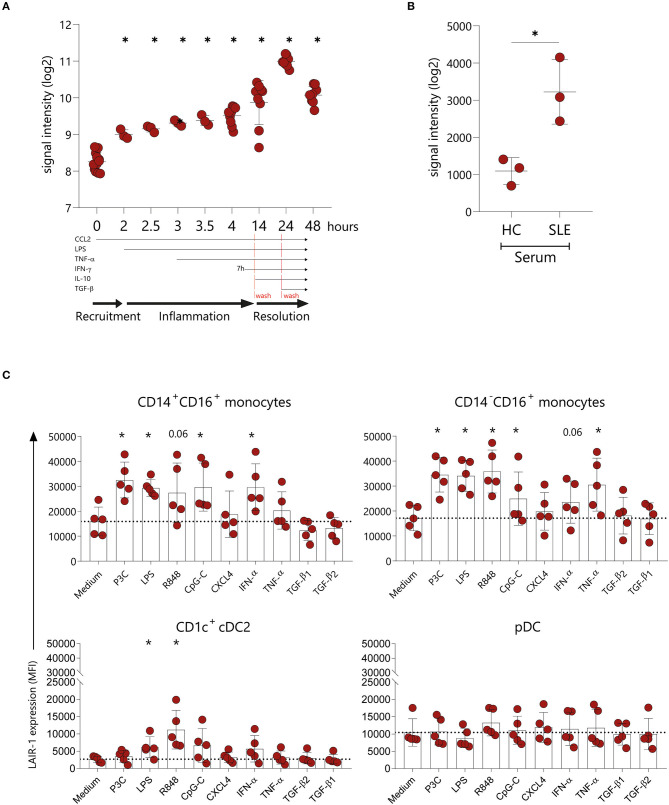
LAIR-1 is upregulated upon inflammatory triggers. LAIR-1 gene expression was determined by array profiling and retrieved from publicly available datasets. **(A)** Kinetics of the *LAIR1* expression in human monocytes from an *in vitro* model of inflammation (GSE47122). Results are represented as mean with SD. Differences were considered statistically significant when *p < 0.05 vs. time-point 0 condition (one-way ANOVA test). **(B)** *LAIR1* expression in healthy blood monocytes exposed during 6 h to 20% serum from healthy controls (HC) or newly diagnosed, untreated systemic lupus erythematosus (SLE) patients (GSE46920). Results are represented as mean with SD. Differences were considered statistically significant when *p* < 0.05 (Unpaired *t*-test) **(C)** LAIR-1 expression [represented as median fluorescence intensity (MFI)] on PBMCs stimulated overnight with different TLR agonists, cytokines and chemokines was assessed by flow cytometry on CD14^+^CD16^+^and CD14^−^CD16^+^ monocytes subpopulations as well as on CD1c^+^ DCs and pDCs. Results are represented as mean with SD. Statistically significant differences were considered when *p < 0.05 vs. medium condition (Friedman's test).

The actual dynamic of LAIR-1 expression on monocytes under an inflammatory response remains unclear. Therefore, we made use of available array profiling data from Italiani et al. [GSE47122] (25) to determine LAIR-1 expression (RNA) kinetics in monocytes on the recruitment, inflammatory and resolution phase of the immune response mimicked *in vitro*. In this model, *LAIR1* was rapidly upregulated 2 h after CCL2 chemoattractant treatment, and these expression levels were maintained under inflammation triggered by LPS and TNF-α (inflammatory phase). *LAIR1* expression was further increased upon treatment with IFN-γ (time point 14 h). Furthermore, during the initial step of the resolution phase, the addition of IL-10 led to the highest *LAIR1* expression. In the final stage of resolution, TGF-β addition resulted on downregulation of *LAIR1* expression to the levels found during the inflammatory phase (Figure 2A). Since SLE is a known chronic inflammatory disease and the soluble mediators present in SLE patients' serum are able to induce and perpetuate an inflammatory response (26–28), we next made use of the model from Rodriguez-Pla et al. [GSE46920] (29), in which HC monocytes cultured in the presence of SLE serum, exhibited higher *LAIR1* levels when compared to HC serum-treated monocytes (Figure 2B).

In order to further investigate the regulation of LAIR-1 expression on the different monocytes subsets and DCs subpopulations, we isolated PBMCs and stimulated them with different TLR agonists, chemokine and cytokines. Monocytes were identified based on HLA-DR and CD11c expression and even though CD16 is upregulated on monocytes in culture [Supplementary Figure 3A and as observed by others (30)], it was possible to identify two different subsets of monocytes (CD14^+^CD16^+^ and CD14^−^CD16^+^ monocytes). On both monocyte populations, LAIR-1 expression increased upon TLR2/1, TLR4, TLR7/8, TLR9, or IFN-α stimulation, compared to medium alone. Remarkably, TNF-α stimulation induced LAIR-1 expression only on the CD14^−^CD16^+^ monocyte population (Figure 2C, Supplementary Figure 3B). On CD1c^+^ cDC2, LAIR-1 expression only increased after TLR4 and TLR7/8 stimulation (Figure 2C, Supplementary Figure 3B). On pDC, LAIR-1 expression was stable regardless of stimulation (Figure 2C, Supplementary Figure 3B). Furthermore, stimulation with CXCL4 or TGF-β did not modulate LAIR-1 expression on any cell type (Figure 2C, Supplementary Figure 3B). Thus, *in vitro*, inflammatory mediators lead to LAIR-1 upregulation on monocytes and cDC2s.

### Monocyte Function Is Modulated *in vitro* via LAIR-1

**Figure 3:**
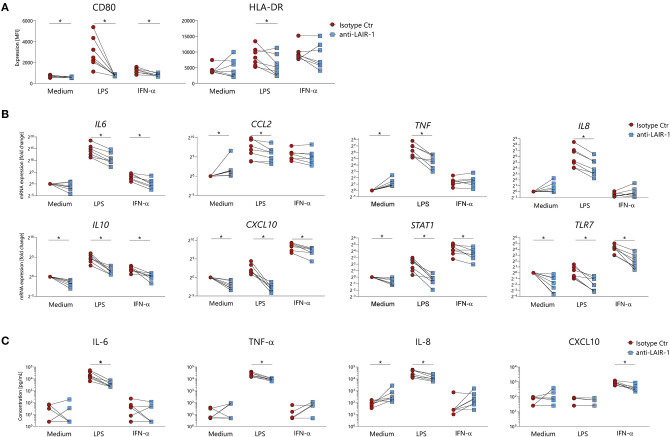
LAIR-1 triggering modulates monocytes activation upon *in vitro* stimulation. **(A)** PBMCs were pre-treated with anti-LAIR-1 agonist (Dx26) or isotype control (2 h) and stimulated overnight with TLR4 agonist- LPS or IFN-α and the expression of CD80 and HLA-DR was determined on gated monocytes, using flow cytometry. **(B,C)** MACS purified monocytes were pre-treated (2 h) with anti-LAIR-1 agonist (Dx26) or isotype control and stimulated 5 h with TLR4 agonist- LPS or IFN-α. **(B)** *IL6, CXCL10, TNF, TLR7, IL8, IL10, CCL2*, and *STAT1* gene expression was evaluated by qRT-PCR and **(C)** protein production of IL-6, TNF-α, IL-8, and CXCL10 was measured by ELISA. Results are represented as paired samples. Statistically significant differences were considered when *p < 0.05 (Wilcoxon's test).

In order to understand the role of LAIR-1 in the regulation of monocyte function, LAIR-1 was crosslinked with a specific anti-LAIR-1 agonistic antibody (clone Dx26) prior to LPS (TLR4 ligand) or IFN-α stimulation. In cultured PBMCs, LAIR-1 signaling prevented LPS or IFN-α induced upregulation of the co-stimulator molecule CD80 and LPS induced HLA-DR expression on monocytes (Figure 3A). The expression of other markers, such as CD86, HLA class I (HLA-ABC) and activating collagen receptor osteoclast-associated immunoglobulin-like receptor (OSCAR) was not modulated upon stimulation. Of note, LAIR-1 expression was downregulated upon LAIR-1 engagement, most likely due to receptor internalization (Supplementary Figure 4).

In purified monocytes, LAIR-1 ligation inhibited LPS induced *IL-6, TNF, IL8, CCL2, CXCL10, TLR7, IL10*, and *STAT1* mRNA expression (Figure 3B). In line with mRNA expression, LPS induced IL-6, TNF-α, and IL-8 protein production was also inhibited by LAIR-1 triggering (Figure 3C). Additionally, LAIR-1 signaling inhibited IFN-α induced *IL6, CXCL10, TLR7, IL10*, and *STAT1* mRNA expression (Figure 3B). For CXCL10 this was confirmed at protein level (Figure 3C). In unstimulated cells, LAIR-1 activation led to decreased *CXCL10, TLR7, IL10*, and *STAT1* gene expression while *TNF* and *CCL2* gene expression was increased. Furthermore, LAIR-1 ligation resulted in increased IL-8 protein production (Figures 3B,C). Taken together, LAIR-1 ligation inhibits both TLR4 activating signals and IFN mediated responses *in vitro*.

### LAIR-1 Is Downregulated on GM-CSF and IFN-γ Monocyte-Derived Macrophages and Monocyte Derived-Dendritic Cells

**Figure 4:**
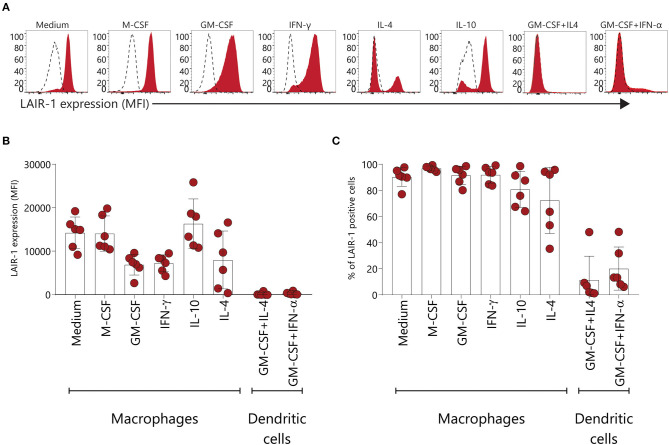
LAIR-1 is downregulated on GM-CSF and IFN-γ monocyte-derived macrophages and monocyte derived-dendritic cells. **(A)** Representative histogram of LAIR-1 expression assessed on different types of *in vitro* monocyte-derived macrophages and dendritic cells using flow cytometry. **(B)** Quantification of LAIR-1 expression represented as median fluorescence intensity (MFI). **(C)** The percentage (%) of cells expressing LAIR-1 is displayed. Results are represented as mean with SD.

Under inflammatory conditions, monocytes are attracted from the circulation to injured tissues and once arrived, monocytes can enter macrophage or dendritic cell reprograming, depending on the micro-environment (31). Here we assessed LAIR-1 expression on monocyte-derived macrophages and dendritic cells differentiated *in vitro*. LAIR-1 expression was downregulated on GM-CSF and IFN-γ derived macrophages, while on M-CSF and IL-10 derived macrophages LAIR-1 expression was maintained (Figures 4A,B). Moreover, on IL-4 and IL-10 derived macrophages LAIR-1 expression was downregulated on a subset of cells (Figures 4A,B). LAIR-1 was profoundly downregulated on monocyte-derived dendritic cells differentiated in the presence of GM-CSF combined with IL-4 or combined with IFN-α, with only an average of 11.5 and 21.1% of LAIR-1- expressing cells, respectively (Figures 4B,C). Thereby, these results demonstrate that monocyte reprogramming toward macrophages or DCs critically regulates LAIR-1 expression; while GM-CSF and IFN-γ differentiated macrophages (M1) and moDCs downregulate LAIR-1 expression, M-CSF, IL-10 and IL-4 differentiated macrophages (M2) maintain in great part LAIR-1 expression.

### LAIR-1 Impacts Monocyte-Derived Dendritic Cell Differentiation

**Figure 5:**
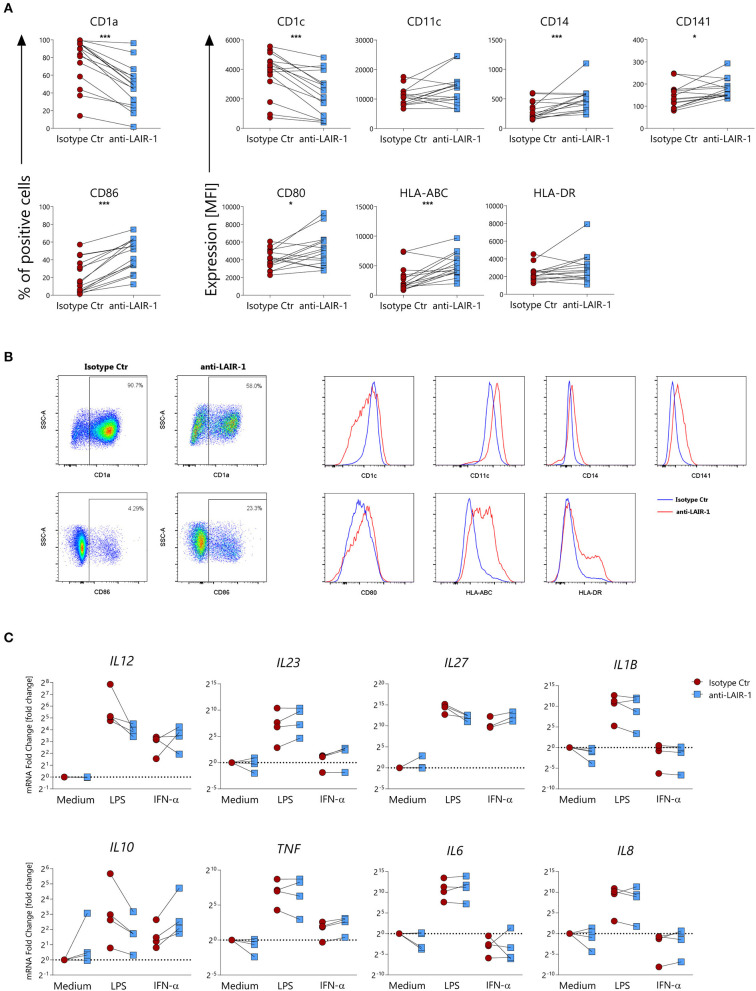
LAIR-1 activation during differentiation of monocyte-derived dendritic cells results in phenotypic and cytokine profile alterations. **(A)** Purified monocytes were differentiated for 6 days into monocyte-derived dendritic cells using GM-CSF and IL-4 in the presence of anti-LAIR-1 agonist (Dx26) or isotype control and the expression of CD1a, CD1c, CD11c, CD14, CD141, CD86, CD80, HLA-ABC, and HLA-DR was assessed by flow cytometry. Quantification is shown as percentage (%) of positive cells or median fluorescence intensity (MFI). **(B)** Representative plots or histograms are shown. **(C)** Monocyte derived dendritic cells differentiated in the presence of anti-LAIR-1 agonist (Dx26) or isotype control were stimulated during 4 h with TLR4 agonist- LPS or IFN-α and the *IL12A, IL23A, IL27A, IL1B, IL10, TNF, IL6, IL8* gene expression was evaluated by qRT-PCR. Results are represented as paired samples. Statistically significant differences were considered when *p < 0.05, ***p < 0.001 (Wilcoxon's test).

To examine the contribution of LAIR-1 activation on monocyte derived dendritic cells differentiation, we generated dendritic cells from monocytes (moDCs) with GM-CSF and IL-4 in the presence of plate bound anti-LAIR1 antibody (clone Dx26). LAIR-1 ligation during moDCs differentiation resulted in a lower percentage CD1a^+^ or CD1c expressing cells when compared to the isotype control condition. On the other hand, LAIR-1 ligation resulted in a higher percentage of CD86^+^ cells and increased CD14, CD141, HLA-ABC, and HLA-DR expression. LAIR-1 ligation did not affect CD11c and CD80 expression (Figures 5A,B). Thus, LAIR-1 ligation during moDC differentiation, clearly alters their phenotype with potential impact on their function.

We next sought to understand whether moDC differentiated in the presence of LAIR-1 ligation responded differently to subsequent TLR4- ligand (LPS) or IFN-α stimulation. After 6 days of differentiation, moDCs were harvested and further stimulated with LPS or IFN-α. moDCs differentiated in the presence of LAIR-1 agonist and stimulated with LPS expressed lower mRNA levels of *IL12A, IL27A, IL1B, IL8*, and *IL10* but showed higher expression levels of *IL23* (Figure 5C). On the other hand, IFN-α stimulation of moDCs differentiated in the presence of LAIR-1 agonist resulted in increased gene expression levels of *IL27A, IL10*, and *TNF* (Figure 5C). Hence, LAIR-1 ligation during moDC differentiation changes the response to TLR4 and IFN-α stimulation.

### LAIR-1 Dynamics During Inflammation *in vivo*

**Figure 6:**
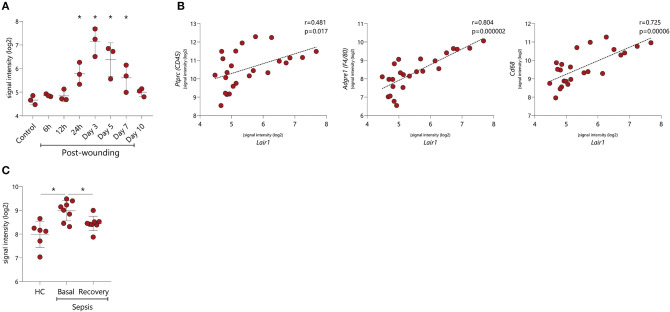
LAIR-1 *in vivo* dynamics during inflammation. LAIR-1 gene expression was determined by array profiling and retrieved from publicly available datasets. **(A)** *Lair1* expression in mouse skin samples recovered from injury up to 10 days following wounding inflicted via punch biopsy (GSE23006). Results are represented as mean with SD. Differences were considered statistically significant when *p < 0.05 vs. control sample (one-way ANOVA test). **(B)** Correlation between *Lair1* expression and *Ptprc* (CD45), *Adgre1* (F4/80), and *Cd68* expression in mouse skin samples recovered from injury up to 10 days following wounding inflicted via punch biopsy (GSE23006). Correlations were assessed by the Pearson's correlation coefficient test. **(C)** LAIR-1 expression on peripheral blood monocytes from gram-negative sepsis patients during sepsis and following their recovery (GSE46955). Results are represented as mean with SD. Differences were considered statistically significant when *p < 0.05 (HC vs. sepsis basal and HC vs. sepsis recovery Unpaired *t*-test, sepsis basal vs. sepsis recovery Paired *t*-test).

Our *in vitro* data points toward an upregulation of LAIR-1 upon stimulation with several inflammatory mediators, however, the actual *in vivo* LAIR-1 regulation remains to unveil. Wound healing in mice is a conventional *in vivo* model to explore dynamics of inflammation during tissue repair. In the array profiling performed by Chen L et al. in this model [GSE23006] (32), we found that *Lair1* expression was unaltered during the first 12 h after the wound was induced, but after 24 h, *Lair1* expression was upregulated, with the highest *Lair1* expression being detected 3 days after injury. *Lair1* expression decreased at day 5 and normalized to the level of unwounded tissue at day 10 (Figure 6A). We next investigated a potential relation between the LAIR-1 dynamics of expression in this model with immune infiltration. Indeed, we observed a minor correlation between *Lair1* and *Ptprc* (CD45 gene) but a strong correlation between *Lair1* and macrophage markers like *Adgre1* (F4/80 gene) and *Cd68* (Figure 6B). Therefore, *Lair1* expression during wound-healing in this model may be related with macrophage infiltration.

Human sepsis is an example of a dysregulated inflammatory response to infection (33) and represents an interesting model to comprehend LAIR-1 dynamics *in vivo*. Array profiling data, on *ex vivo* isolated blood monocytes from HC and gram-negative sepsis patients during sepsis (basal) and following their recovery (recovery) were published by Shalova et al. [GSE46955] (34). *LAIR1* expression was higher in patient monocytes during sepsis compared to HC and was restored to HC levels after the recovery process (Figure 6C). These data indicate that the expression of LAIR-1 is dynamic and varies during the different phases of inflammation and resolution of the immune response.

## Discussion

It has been proposed that the interaction between LAIR-1 and its ligands should be critically regulated to ensure a balanced immune response. Differential expression of LAIR-1 on B cells, T cells, DCs and neutrophils was previously reported (7), pointing toward the importance of the expression levels for the regulation of LAIR-1-mediated inhibition.

Monocytes and DCs have a high complexity and heterogeneity (35, 36). In line with this, LAIR-1 is differently expressed among the different circulating monocytes subsets. Intermediate monocytes highly express LAIR-1 when compared to classical and non-classical monocytes. This differential expression could be related with the actual role of each monocyte subset in inflammation. For instance, the intermediate monocytes subset expresses the highest levels of antigen presentation-related molecules and was shown to produce higher amounts of TNF-α, IL-1β, IL-6, and CCL3 upon TLR stimulation. In addition, this cell subset is often increased in many inflammatory conditions (36–39). Thus, high levels of LAIR-1 expressed by this cell subset may reflect the importance of regulation of inflammation to return to homeostasis. In line, *in vitro* we demonstrate that stimulation with inflammatory cues, such as serum of SLE patients, TLR ligands, IFN-α or TNF-α, leads to LAIR-1 upregulation on monocytes and CD1c^+^ cDC2s. This indicates, that in inflammatory conditions there might be a need for LAIR-1 upregulation to interact with its ligands in order to readily tune down the immune response. Of note, LAIR-1 upregulation mediated by IL-10 in the initial phase of the resolution phase can be important to inhibit the ongoing inflammatory process, however maintaining high levels of LAIR-1 could lead to an exacerbated inhibitory response, with detrimental effects. In this context, TGF-β seems to be important to return LAIR-1 expression back to homeostatic levels. Interestingly, stimulation with TGF-β1 and TGF-β2 alone did not impact LAIR-1 expression in monocytes or DCs.

In pDCs, we confirmed the high levels of LAIR-1, reported before (16), while the expression on CD1c^+^ cDC2 is comparably lower. Whether LAIR-1 is maintained in tissue resident cells is yet unclear. Here we show that LAIR-1 is highly expressed on tissue macrophages and tissue CD14^+^ cells present in skin, indicating that LAIR-1 might be an important mediator maintaining immune tolerance in peripheral tissues, especially in the presence of a high abundance of collagen. Interestingly, we found that circulating CD141^+^ cDC1 do not express LAIR-1. Likewise, it has already been shown that cDC1s also lack the expression of other inhibitory receptors such as ILT2 and that PD-L1 is low expressed (40). This demonstrates that CD141^+^ cDC1 display a different profile of inhibitory receptors compared with cDC2, and are not regulated via LAIR-1 in circulation or skin.

In monocytes, we also show that LAIR-1 regulates the expression of CD80 and HLA-DR, which indicates a potential importance for LAIR-1 in balancing antigen-presenting cell—T cell interaction. Furthermore, LAIR-1 triggering in monocytes modulates LPS-TLR4 and IFN-α mediated responses. All together, these findings implicate that LAIR-1 is expressed under inflammatory conditions and it is able to modulate immune responses to multiple activating cues. As a remark, we observed induction of CCL2 and IL-8 upon LAIR-1 antibody stimulation in unstimulated cells, which could be due to FC receptor mediated signals, which cannot be completely excluded in these experiments.

Macrophages are very plastic cell types that can be found in all tissues, displaying an enormous functional diversity as they play diverse roles in the development, homeostasis, tissue repair and immunity (41). We show that LAIR-1 expression on *in vitro* GM-CSF and IFN-γ differentiated macrophages (M1 macrophages) is low in line with similar observations in IFN-γ or IFN-γ+LPS stimulated THP-1-derived macrophages by Jin et al. (42). The low LAIR-1 levels on this macrophage type may contribute to their inflammatory profile. In line with this, LAIR-1 expression is maintained on M-CSF and IL-10 differentiated macrophages, associated with wound healing/immunoregulatory M2 macrophages (43, 44), which have an anti-inflammatory role. Consequently, retaining LAIR-1 expression might be beneficial for their immunosuppressive function.

MoDCs differentiated in the presence of LAIR-1 ligation have low CD1a and CD1c expression, but higher levels of CD86 (co-stimulatory molecule), CD14, HLA-ABC and HLA-DR molecules. These results are in line with previous reports indicating that LAIR-1 engagement can regulate the differentiation of monocytes into DCs with GM-CSF (45) and that LAIR-1 ligand C1q and C1 complexes are able to inhibit the differentiation of monocytes into DCs (11). Of note, the CD1a negative moDCs were previously shown to produce lower amounts of IL-12 upon stimulation and have less capacity to polarize T cells to a Th1 phenotype (46). The heterogeneity within moDC cultures is elegantly discussed by Sander et al. (47). Additionally, we also showed that LAIR-1 activation during moDC differentiation alters the response to TLR4 and IFN-α stimulation, whereas DCs differentiated in the presence of LAIR-1 ligation, have a lower inflammatory response to TLR4 activation, IFN-α stimulation results in an increased inflammatory response. On one hand, these cells can actively participate on the host defense against viruses (48), but on the other hand may play a potential role in the perpetuation of type I interferon-mediated autoimmune diseases (49).

Since an exacerbated inflammatory response might be potentially harmful, the control of the pro-inflammatory mechanisms by an anti-inflammatory counterbalance is an important protective process against further enhancement of inflammation (50, 51). Our different *in vitro* and *in vivo* models indicate that inflammation leads to an upregulation of LAIR-1, as observed here in monocytes from sepsis patients, but also on circulating monocytes in acute myocardial infarction, rheumatoid arthritis and liver cirrhosis (52–54). The upregulation of LAIR-1 during inflammation, for instance mediated by TLR or IFN signals, will facilitate its inhibitory signals. As shown here, this ranges from controlling the production of classical inflammatory cytokines (i.e., IL-6, TNF-α, or IL-8), but also IL-10 or IFN inducible proteins as CXCL10.

In conclusion, we show that LAIR-1 is broadly expressed on different monocyte subsets and macrophages, not only in circulation but also in tissue. Under inflammatory conditions LAIR-1 is upregulated and upon ligation its intrinsic inhibitory capacity is functional, and is able to reprogram monocyte derived DC function. Thereby, our data indicate that LAIR-1 is a potentially targetable receptor to damp the immune responses in inflammatory conditions.

## Data Availability Statement

The raw data supporting the conclusions of this article will be made available by the authors, without undue reservation, to any qualified researcher.

## Ethics Statement

This study was reviewed and approved by the Medical Ethical Committee of the University Medical Centre Utrecht. All the participants provided their written informed consent to participate in this study in accordance with the Declaration of Helsinki.

## Author Contributions

TC, SG, MP, TR, WM, and LM were involved in study conception and design of the experiments. TC and BG carried out the experiments. Analysis and interpretation of data was performed by TC, SG, MP, TR, WM, and LM. All authors were involved in drafting the manuscript or revising it critically, and all authors approved the final version.

## Conflict of Interest

LM has regular interaction with pharmaceutical and other industrial partners. She has not received personal fees or other personal benefits. LM's institute has received funding for investigator-initiated studies from Nextcure, Boehringer Ingelheim, Ono Pharmaceuticals, Ablynx and Janssen. LM received minor funding for consultation from Novo Nordisk, Biogen and Boehringer Ingelheim. The remaining authors declare that the research was conducted in the absence of any commercial or financial relationships that could be construed as a potential conflict of interest.
