# Supplementary material for: Leukocyte Associated Immunoglobulin Like Receptor 1 Regulation and Function on Monocytes and Dendritic Cells During Inflammation
Source: Front Immunol. 2020 Aug 19;11:1793. doi: 10.3389/fimmu.2020.01793 (PMC7466540; doi:10.3389/fimmu.2020.01793)
Supplement: Supplementary file 1 [file Data_Sheet_1.pdf]

Supplementary Material

1 Supplementary Figures and Tables

1.1 Supplementary Figures

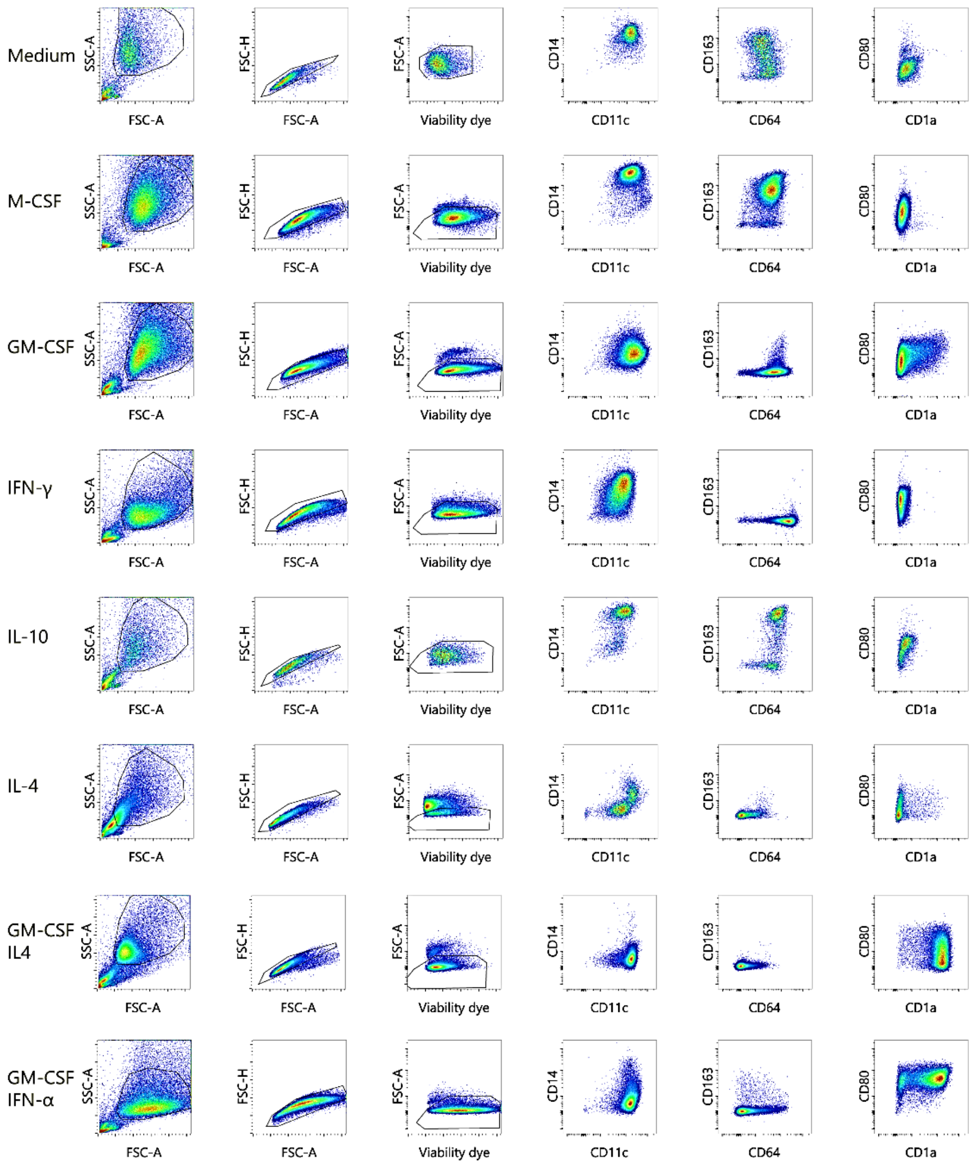

Supplementary Figure 1. Analysis strategy and phenotype of monocyte-derived macrophages and dendritic cells.

Flow cytometry gating strategy analysis of the different types of *in vitro* monocyte-derived macrophages and dendritic cells and representative dot plots for the expression of CD14, CD11c, CD163, CD64, CD1a and CD80, used to assess differentiation.

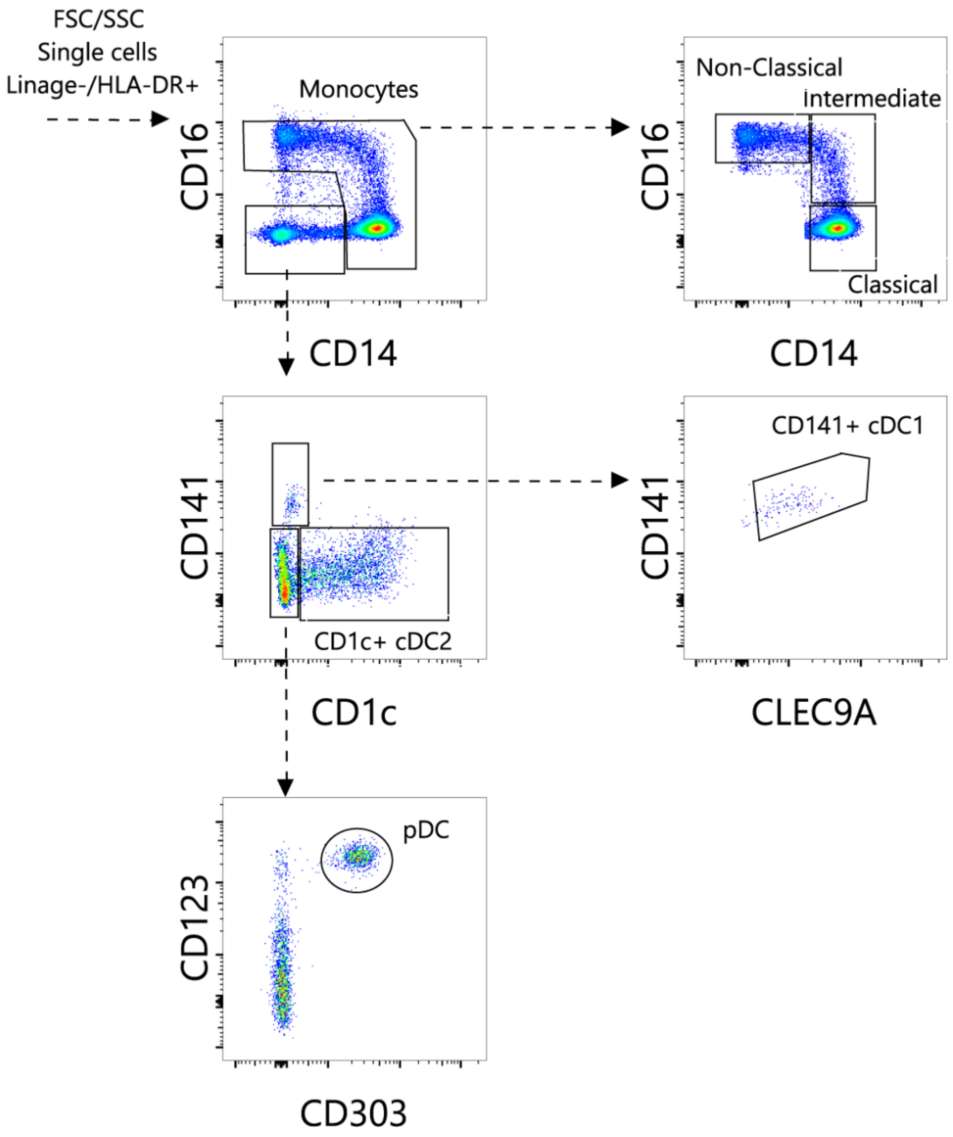

Supplementary Figure 2. Analysis strategy to identify classical, intermediate and non-classical monocytes as well as CD1c^{+}cDC1s, CD141^{+}cDC2s and pDCs.

Flow cytometry gating strategy analysis to identify classical, intermediate and non-classical monocytes as well as CD1c^{+}cDC1s, CD141^{+}cDC2s and pDCs on peripheral blood mononuclear cells (PBMC).

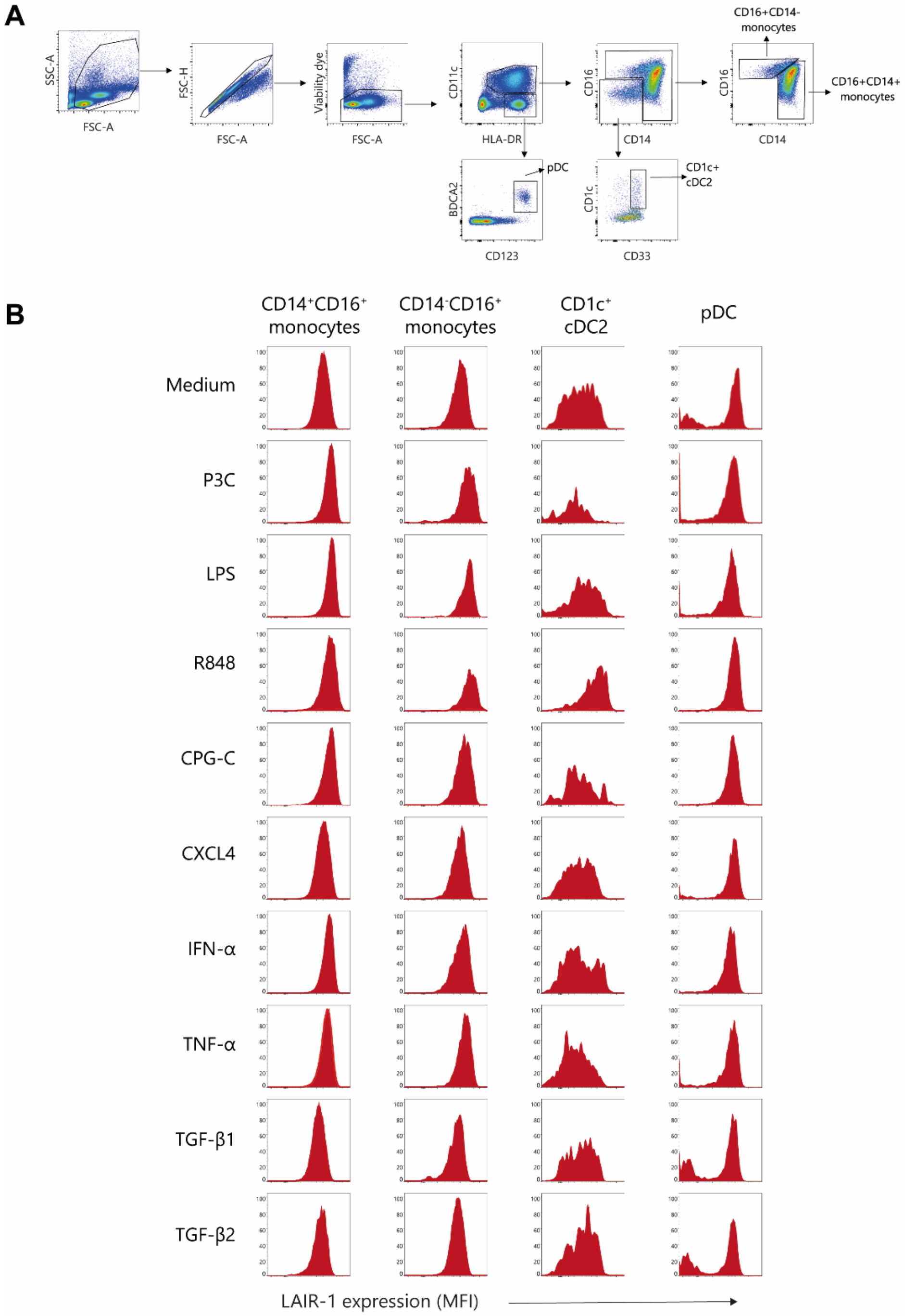

Supplementary Figure 3. Analysis strategy to determine LAIR-1 expression on stimulated PBMCs.

(A) Flow cytometry gating strategy analysis to identify CD14^{+}CD16^{+} and CD14^{-}CD16^{+} monocytes subpopulations as well as CD1c^{+} cDC2 and pDC on PBMCs stimulated with different TLR agonists, cytokines and chemokines. **(B)** Representative histogram of LAIR-1 expression on stimulated CD14^{+}CD16^{+} and CD14^{-}CD16^{+} monocytes, as well as on CD1c^{+} cDC2 and pDC.

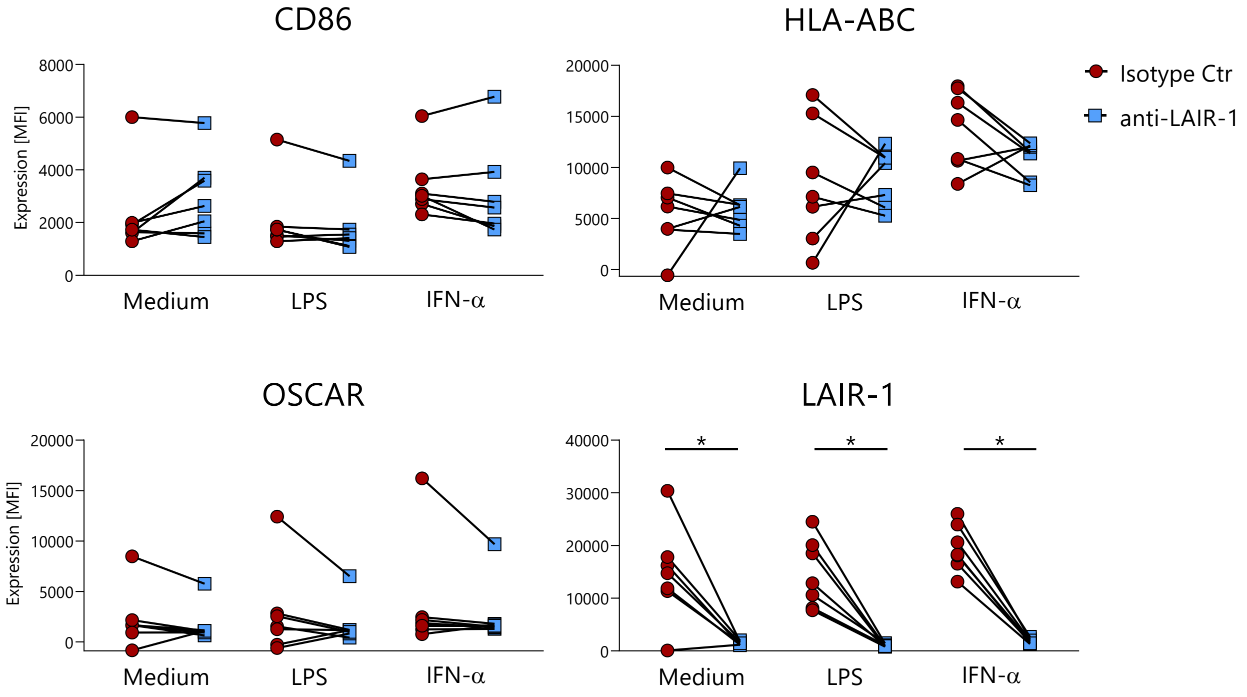

Supplementary Figure 4. CD86, HLA-ABC, OSCAR and LAIR-1 expression on monocytes upon LAIR-1 triggering.

Peripheral blood mononuclear cells were pre-treated with anti-LAIR-1 agonist (Dx26) or isotype control (2 h) and stimulated overnight with TLR4 agonist- LPS or IFNα and the expression of CD86, HLA-ABC, Osteoclast-associated immunoglobulin-like receptor (OSCAR) and LAIR-1 was determined on gated monocytes, using flow cytometry. Results are represented as paired samples. Statistically significant differences were considered when $*p<0.05$ (Wilcoxon's test).

1.2 Supplementary tables

Supplementary Table 1: Antibody panels used on the flow cytometry stainings.

LAIR-1 expression on freshly isolated PBMCs (Fig. 1A-B)

Target	Label	Manufacturer	Catalogue nr.	Clone
CD3	AF700	Biolegend	300424	UCHT1
CD19	AF700	eBioscience	56-0199-42	HIB19
CD56	AF700	BD Pharmingen	557919	B159
CD1c	BV421	Sony Biotechnology	2257630	L161
CD14	APC-eFluor	eBioscience	47-0149-42	61D3
CD16	V500	BD Horizon	561394	3G8
CD123	PE-Cy7	Biolegend	306010	6H6
CD141	BV711	BD Horizon	563155	1A4
CLEC9A	FITC	Miltenyi Biotec	130-097-403	8F9
CD303	PerCP-Cy5.5	Sony Biotechnology	2371050	201A
HLA-DR	BV605	BD Horizon	562845	G46-6
LAIR-1	PE	BD Pharmingen	550811	DX26
Isotype control IgG1	PE	BD Pharmingen	555749	MOPC-21

LAIR-1 expression on skin (Fig. 1D)

**Target**	**Label**	**Manufacturer**	**Catalogue nr.**	**Clone**
CD90	PECy7	Biolegend	328124	3.9
CD1c	BV421	Biolegend	331526	L161
CD45 PerCP	PerCP-Cy5.5	Biolegend	304026	IT2.2
HLA-DR	BV605	BD Biosciences	562845	3G8
CD141	BV711	BD Biosciences	563155	6H6
CD14	BV785	Biolegend	301840	AC144
CD1a	BV510	BD Biosciences	563481	M5E2
CD11c	AF700	eBioscience	56-0116-42	G46-6
LAIR-1	PE	BD Pharmingen	550811	P67.6
Isotype control IgG1	PE	BD Pharmingen	555749	DX26
Fixable Viability Dye	eFluor 780	Bioscience<br>Thermo Fisher Scientific	65-0865-14	-

* autofluorescence detected on FITC channel was used to define tissue macrophages.

LAIR-1 expression on stimulated PBMC (Fig. 2C)

Target	Label	Manufacturer	Catalogue nr.	Clone
CD11c	AF700	eBioscience	56-0116-42	3.9
CD1c	BV421	Sony Biotechnology	2257630	L161
CD86	BV605	Biolegend	305430	IT2.2
CD16	V500	BD Horizon	561394	3G8
CD123	PE-Cy7	Biolegend	306010	6H6
BDCA2	APC	Miltenyi Biotec	130-090-905	AC144
CD14	BV785	Biolegend	301840	M5E2
HLA-DR	FITC	BD Pharmingen	555811	G46-6
CD33	PerCP-Cy5.5	BD Biosciences	333146	P67.6
LAIR-1	PE	BD Pharmingen	550811	DX26
Fixable Viability Dye	eFluor 780	Bioscience<br>Thermo Fisher Scientific	65-0865-14	-
Isotype control IgG1	PE	BD Pharmingen	555749	MOPC-21

LAIR-1 mediated inhibition on PBMC (Fig. 3A)

Target	Label	Manufacturer	Catalogue nr.	Clone
CD14	BV785	Biolegend	301840	M5E2
CD16	V500	BD Biosciences	561394	3G8
HLA-DR	BV605	BD Biosciences	562845	G46-6
CD45	PerCP	Biolegend	304026	HI30
CD86	PB	Biolegend	2127090	IT2.2
OSCAR	PE	Beckman Coulter	A24987	11.1CN5
HLA-ABC	PE-Cy7	BD Biosciences	561349	G46-2.6
LAIR1	AF647	Sony Biotechnology	2314010	NKTA255
CD80	FITC	BD Pharmingen	557226	L307.4
Fixable Viability Dye	eFluor 780	Bioscience<br>Thermo Fisher Scientific	65-0865-14	

LAIR-1 expression on monocyte derived- macrophages / dendritic cells (Fig 4A-B)

**Target**	**Label**	**Manufacturer**	**Catalogue nr.**	**Clone**
CD1a	eF450	eBioscience	48-0019	HI149
CD80	APC-H7	BD Pharmingen	561134	L307.4
CD64	PE-Cy7	BD Pharmingen	561191	10.1
CD163	APC	eBioscience	17-1639-42	eBioGHI/61
LAIR1	PE	BD Pharmingen	550811	DX26
CD11c	FITC	Sony Biotechnology	2286070	Bu15
CD14	PerCPCy55.5	Biolegend	325622	HCD14
CD1a	eF450	eBioscience	48-0019	HI149
CD80	APC-H7	BD Pharmingen	561134	L307.4
CD64	PE-Cy7	BD Pharmingen	561191	10.1
LAIR1	PE	BD Pharmingen	550811	DX26
Isotype control IgG1	PE	BD Pharmingen	555749	MOPC-21
Fixable Viability Dye	eFluor 506	Bioscience<br>Thermo Fisher Scientific	65-0866-14	-

Monocyte derived- dendritic cells phenotype (Fig. 5A-B)

Target	Label	Manufacturer	Catalogue nr.	Clone
CD14	PerCP-Cy5.5	Biolegend	301824	M5E2
CD11c	AF700	eBioscience	56-0116-42	3.9
CD1a	BV510	BD Biosciences	563481	HI149
CD1c (BDCA1)	BV421	Biolegend	331526	L161
LAIR1	AF647	Sony Biotechnology	2314010	NKTA255
CD86	BV605	Biolegend	305430	IT2.2
HLA DR (MHCII)	BV711	BD Biosciences	563696	G46-6
HLA ABC (MHCI)	PE-Cy7	BD Biosciences	561349	G46-2.6
OSCAR	PE	Beckman Coulter	A24987	11.1CN5
CD80	APC-H7	BD Pharmingen	561134	L307.4
CD141 (BDCA3)	FITC	Miltenyi Biotec	130-090-513	AD5-14H12

Supplementary table 2. Primer sequences used for RT-qPCR

**Gene**	**Foward Sequence 5’ - 3’**	**Reverse Sequence 5’ - 3’**
B2M	GATGAGTATGCCTGCCGTGT	TGCGGCATCTTCAAACCTCC
RPL13A	CCTGGAGGAGAAGAGGAAAGAGA	TTGAGGACCTCTGTGTATTTGTCAA
IL12A	CTCCAGAAGGCCAGACAAAC	AATGGTAAACAGGCCTCCACT
IL23A	GCTTGCAAAGGATCCACCA	TCCGATCCTAGCAGCTTCTCA
IL27A	ATCTCACCTGCCAGGAGTGAA	TGAAGCGTGGTGGAGATGAAG
IL1B	TTTGAGTCTGCCCAGTTCCC	TCAGTTATATCCTGGCCGCC
IL10	GAGGCTACGGCGCTGTCAT	CCACGGCCTTGCTCTTGTT
TNF	GGAGAAGGGTGACCGACTCA	CTGCCCAGACTCGGCAA
IL6	TGCAATAACCACCCCTGACC	TGCGCAGAATGAGATGAGTTG
IL8	TGAGAGTGGACCACACTGCG	TCTCCACAACCCTCTGCACC
TNF	GGAGAAGGGTGACCGACTCA	CTGCCCAGACTCGGCAA
IL8	TGAGAGTGGACCACACTGCG	TCTCCACAACCCTCTGCACC
CXCL10	TGAAATTATTCCTGCAAGCCAA	CAGACATCTCTTCTCACCCTTCTTT
CCL2	TCTGTGCCTGCTGCTCATAG	GGGCATTGATTGCATCTGGC
TLR7	CAAGAAAGTTGATGCTATTGGGC	TGGTTGAAGAGAGCAGAGCA
IL10	GAGGCTACGGCGCTGTCAT	CCACGGCCTTGCTCTTGTT
STAT1	ATGGCAGTCTGGCGGCTGAATT	CCAAACCAGGCTGGCACAATTG
